# The Use of De-Vulcanized Recycled Rubber in the Modification of Road Bitumen

**DOI:** 10.3390/ma13214864

**Published:** 2020-10-30

**Authors:** Barbara Gawdzik, Tadeusz Matynia, Krzysztof Błażejowski

**Affiliations:** 1 Department of Polymer Chemistry, Institute of Chemical Sciences, Faculty of Chemistry Maria Curie-Sklodowska University in Lublin, ul. Gliniana 33, 20-614 Lublin, Poland; tmatynia@poczta.umcs.lublin.pl; 2 Orlen-Asfalt, ul. Chemików 7, 09-411 Płock, Poland; krzysztof.blazejowski@orlen.pl

**Keywords:** bitumen, chemical modification, recycled rubber, de-vulcanization, SBS

## Abstract

Rubber from recycled car tires and styrene-butadiene-styrene (SBS) were used for the chemical modification of commercially available road bitumen 50/70 (EN 12591). The modification process began with the addition of rubber into asphalt and heating the whole amount at the temperature of 190 °C or 220 °C. Under such conditions, de-vulcanization of rubber took place. Next, SBS and sulfur as a cross-linker were added and the heating was continued so that cross-linking of SBS and the de-vulcanized rubber proceeded. In the studies on the influence of rubber concentration on the final properties of asphalt 10% or 15% of rubber was considered. Chemical modification reactions were performed within 2, 4, and 8 h. The results showed that both the modification at 190 °C and 220 °C affected the properties of the base asphalt efficiently, although the asphalt modified at 190 °C contained more non-degraded rubber. Increasing the modification time led to dissolution of the rubber crumbs and its de-vulcanization. Bitumens modified in this way are characterized by high storage stabilities. Their behavior at low temperatures also deserves attention.

## 1. Introduction

The increased traffic loads and improvement of pavement working have led to the development of polymer modified bitumens (PMB) during the last few years [1]. There are many categories of asphalt modifiers in use. The most common are thermoplastic elastomers such as styrene-butadiene-styrene (SBS), styrene-isoprene-styrene (SIS), and styrene-ethylene/butylene-styrene (SEBS); different polyolefinic plastomers such as polyethylene (PE), polypropylene (PP), ethylene vinyl acetate (EVA), and crumb rubber [2,3,4,5,6,7]. 

Mostly, 2–6% polymer is added to bitumen. The added polymer intensifies the binder properties and allows for the construction of better quality roads. For example, elastomers can resist permanent deformation over stretching and elastically recover once the load is removed. Simultaneously, the addition of polymers causes a significant increase in production costs and complications related to their storage. The low compatibility between the asphalt and polymer can lead to phase separation when the product is stored at a high temperature without mixing [1]. As a result, the polymer-rich phase concentrates on the top. This upper part exhibits high viscosity and is useless for paving applications.

A much cheaper modifier is rubber, particularly from recycled car tires. It has been shown that, on one hand, bitumen–rubber mixtures reduce distress observed in bituminous pavements, but on the other hand, separation of the asphalt and rubber phases creates problems during storage [8,9]. Another disadvantage is the noticeable increase in viscosity, especially at low temperatures [10,11,12,13]. To reduce the problem of phase separation, Wang [14] proposed using swollen rubber powder. In this form, it allows for the creation of a two-phase continuous mixing system consisting of rubber and bitumen. It was found that the addition of rubber crumbs to the pavement technology is very beneficial as it allows waste tire utilization to solve the problem of, but also improves the performance properties of asphalt pavements including the reduction of street noise [15]. Rodríguez-Fernández et al. [16] reported that the asphalt mixtures with crumb rubber were less susceptible to aging than the conventional polymer modified mixtures. Thodesen et al. [17] tested the properties of the mixture containing 10% of crumb rubber and 1% of SBS polymer as an asphalt modifier. The environmental and economic aspects of using crumb rubber as an asphalt binder are described by Pouranian et al. [18,19]. Generally, the rubber modification is advantageous because it makes asphalt materials tougher and reduces the tendency of surfaces to crack and bleed, improving aggregate retention.

Seghar et al. [20] showed that re-vulcanization of the de-vulcanized rubber with the fresh sample did not significantly affect the quality of the final product. Thus, waste rubber management methods are particularly desirable for the production of pavements with properties corresponding to those of the polymer-modified asphalt. In our previous paper, both recycled rubber and synthetic polybutadiene were used as bitumen modifiers [21]. In this study, the results of bitumen modification using rubber and SBS are presented. The aim of our research was to determine whether the resulting products would have properties comparable to those of polymer modified bitumen (PMB), if recycled rubber was used as a modifier. The influence of rubber concentration, temperature, and modification time on asphalt properties was investigated.

## 2. Experimental

### 2.1. Materials

The asphalt 50/70 (EN 12591) used in this study came from ORLEN Asphalt LTD, Płock (Płock, Poland), while the powdered rubber from recycled tires came from Orzel S.A. Poniatowa, Poland. Synthetic poly(styrene-butadiene-styrene) (SBS) was purchased from Brenntag, Poland Ltd (Kędzierzyn-Koźle, Poland). The sulfur donor and paraffin were from POCh (Gliwice, Poland). 

The rubber powder in the presented research had a 0–0.4 mm granulation. The chemical composition of the used bitumen was as follows: saturated hydrocarbons 13.2%, aromatics 53.6%, resins 19.3%, asphaltenes 13.9% (SARA—Saturated hydrocarbons, Aromatics, Resin, Asphaltenes), and crystallizing fraction 0.3% [22]. 

### 2.2. Bitumen Modification

**Figure 1:**
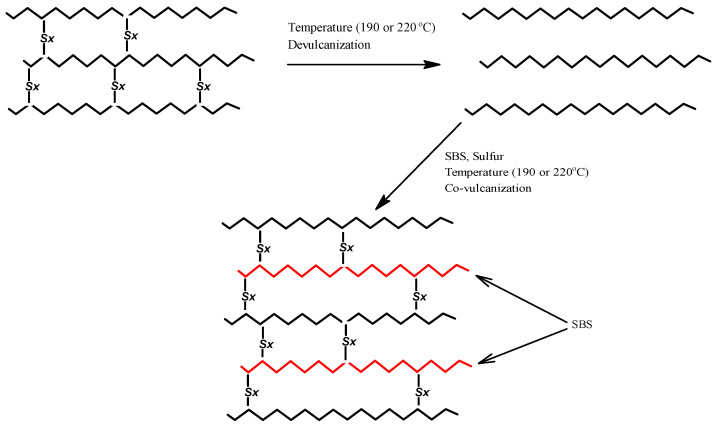
Scheme showing the chemical modification of the studied bitumen.

A total of 760 g of bitumen (88% by weight) and 200 g of crushed rubber (10% by weight) were introduced into a 3000 cm^3^ three-necked round bottom flask, equipped with a mechanical stirrer, thermometer, and reflux condenser. The contents of the flask were heated to 190 °C (or 220 °C), the temperature at which de-vulcanization of the rubber occurs [23,24]. After 1 hour, sulfur and 40 g of SBS (2% by weight) previously swollen with paraffin were added with stirring. Stirring was continued so that the total heating time was: 2, 4, and 8 h. Similar syntheses were performed for the 15% rubber samples (Figure 1). After modification, the bitumen was poured from the flask into molds in which it was cooled to ambient temperature. Each synthesis was repeated three times, and the average product was used in further studies.

### 2.3. Methods Used in the Characterization of Bitumen Properties

#### 2.3.1. Penetration at 25 °C

Penetration (Pen25) at 25 °C was determined according to the standard method (EN 1426). Penetration is the depth at which the needle of the penetrometer is immersed in the asphalt for 5 s under the test at a load of 100 g at 25 °C.

#### 2.3.2. Softening Point

The ring and ball softening point (SP_R&B_) was measured according to EN 1427. SP_R&B_ of asphalt is the conventional temperature at which the asphalt transitions from a viscoelastic to a viscous state. 

This is the temperature at which the asphalt samples in the two rings supporting the steel ball softened, so each ball surrounded by asphalt traveled a distance of 25 ± 0.4 mm.

#### 2.3.3. Fraass Breaking Point

The Fraass breaking point characterizing the behavior of bitumens at low temperatures, was measured according to EN 12593. The breaking point determination consisted in cooling a steel plate with a 0.5 mm thick asphalt layer at a constant speed, and the bending test was applied every minute until asphalt layer cracking was observed.

#### 2.3.4. Softening Point SP_R&B_ Increase

To determine the increase of the SP_R&B_ softening point, a roller thin film oven tester (RTFOT) was used according to EN 1427.

#### 2.3.5. Elastic Recovery at 25 °C

Conventional ductility test was used to determine the elastic recovery of bitumen at 25 °C (EN 13398). It was evaluated as a percentage of recoverable strain measured after elongation.

#### 2.3.6. Stability

Storage stability refers to the tendency of rubber to separate from asphalt and provides an indication of the degree of chemical compatibility between the two individual components. This was determined using aluminum test tubes with a Φ25 mm × 160 mm. The studied samples in the aluminum tubes were heated in the vertical position at 180 °C for 72 h. After cooling, the sample was cut into three parts. For the lower and upper parts, the penetration and softening tests were carried out (difference between penetration top/penetration bottom as delta Pen25, and difference between SPR&B top/SPR&B as delta SPR&B), while the middle part was discorded.

#### 2.3.7. The Mass Change after RTFOT

The mass change after RTFOT (according to EN 12607-1) represents the difference in mass for the samples heated in the thin rolling furnace compared to the mass of the fresh bitumen. 

#### 2.3.8. Cohesion Energies and Maximal Tensile Force

The cohesion energies at 5 °C and 10 °C (according to EN 13589 and EN 13703) and the maximal tensile forces [N] were determined by the force ductility method.

#### 2.3.9. Differential Scanning Calorimetry (DSC)

Differential scanning calorimetry (DSC) measurements were carried out with a Netzsch 204 calorimeter (Selb, Germany) operating in dynamic mode. The dynamic scans were performed at the heating rate of 10 °C/min from room temperature to 300 °C in an argon atmosphere (flow = 20 cm^3^/min). The samples were placed in an aluminum pan with a pierced lid (mass of 40 ± 1 mg). The empty pan was used as a reference sample. The sample masses of 10.0 ± 0.2 mg were used. 

#### 2.3.10. Solid Residue

The solid residual was determined by the gravimetric method.

The values of penetration at 25 °C, softening point (R&B), penetration index (IP) according to EN 12591/Ann.A., elastic recovery, softening point after RTFOT, mass change after RTFOT, and stabilities are the mean of three determinations. The values such as penetration index, delta R&B, and increasing of softening point after RTFOT were calculated based on these mean values. The values of the Fraass breaking point refer to one measurement.

## 3. Results and Discussion

In analogy to the previous studies, modifications of bitumen with the de-vulcanized rubber and SBS in the presence of sulfur were performed at 190 and 220 °C [21,24]. The rubber was added at a concentration of 10 or 15% while the SBS concentration was constant at 2%. To improve the solubility of SBS in asphalt, it was previously swollen in paraffin.

The use of the waste product, recycled rubber as a substitute for synthetic polymers, reduces the costs of preparing the polymer-modified bitumen. Technologies for producing road bitumens with the use of rubber are known, but in the mentioned technologies, rubber is usually used as a cross-linked polymer [25,26]. In this form, rubber does not form a homogeneous mixture with bitumen. 

**Figure 2:**
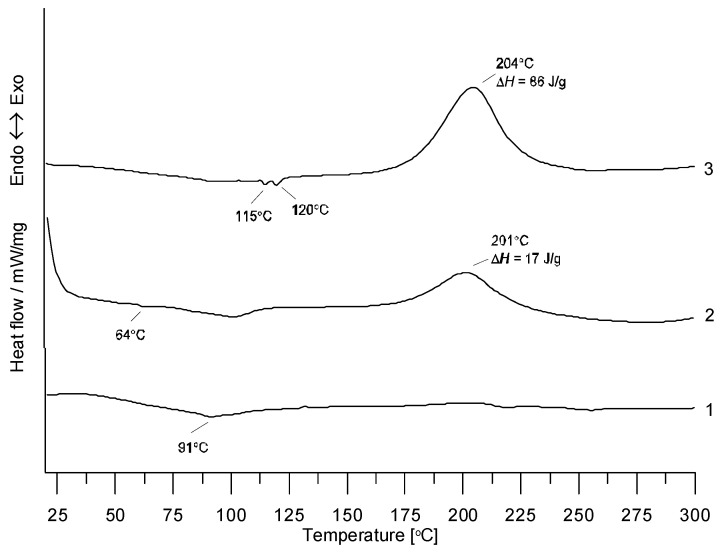
Results of differential scanning calorimetry (DSC) analysis of fresh rubber (1); rubber heated at 190 °C for 2 h to which sulfur was added (2); rubber heated at 190 °C for 2 h to which sulfur and SBS were added (3).

In this study, rubber in the de-vulcanized form was used for the production of PMB. The de-vulcanized rubber can be considered as a thermoplastic polymer. During heating, some polysulfide and disulfide bonds in the three-dimensional network of rubber are converted to the monosulfide bonds, which leads to the formation of uncured rubber [27]. The pre-de-vulcanized waste rubber was then used to create a joint network with SBS in the crosslinking process. The creation of a new polymer network was possible after adding a new dose of sulfur. For better visualization of the process of joint cross-linking of de-vulcanized rubber with SBS in the asphalt environment, this process was carried out without asphalt. Figure 2 presents the results of DSC analysis for the fresh rubber and the rubber samples heated at 190 °C for 2 h to which SBS and sulfur were added. 

From their courses, one can see that for the fresh rubber, no effects were observed, whereas for the samples with a new portion of sulfur, the cross-linking effect was observed. The maximum of an exothermic effect appeared around 200 °C and was even higher in the process of rubber and SBS cross-linking. Small peaks at 115 °C and 120 °C indicate the melting of sulfur in the rhombic and monoclinic allotropic forms. 

When such processes take place in asphalt, a new portion of sulfur gives rise to the formation of sulfur links with different lengths, and additionally, they counteracted high molecular weight phenols present in bitumen (as aromatics and asphaltenes) that have strong polymerization inhibiting properties. Phenols are part of aromatics that are formed during the oxidation process of bitumen [28]. Other compounds are rather nonpolar and can only swell the rubber [12]. These compounds are unlikely to interfere with the polymerization process.

Table 1 and Table 2 present the results of the research on the impact of the time of bitumen modification on the concentration of insoluble residue.

From these data, one can see that the content of insoluble residue depends on both the modification time and the concentration of the rubber. As the modification time increases, the amount of insoluble residue decreases, indicating that the rubber is effectively de-vulcanizing. After 8 h of de-vulcanization at 190 °C, the residue concentrations were 5.0 and 6.4%. The residue contains carbon black and the residues of rubber that were not de-vulcanized. Obviously, for bitumen with a greater rubber concentration, the percentage of residue is larger. The decrease in residue concentration with the increasing reaction time is also visible at 220 °C. At this temperature, the de-vulcanization process runs faster. After the 8 h modification, the residues are 3.3 and 4.5%.

The properties of the modified bitumen are summarized in Table 3 and Table 4.

For the samples heated at 190 °C, the increase in the modification time caused rather insignificant changes of properties (Table 3). For the samples containing 10% of rubber penetration at 25 °C, there were changes in the range of 50.4–47.0 units (0.1 mm). Penetration for bitumens with 15% of rubber had higher and more diverse values (between 54.0 and 64.0 units). The softening point (R&B) of the samples containing 10% of rubber decreased insignificantly with the increasing modification time, whereas for the bitumens containing 15% of rubber, their values increased. However, all obtained values were high. For all modified samples, the Fraass breaking points characterizing bitumen behavior at low temperatures were also impressive. Their values were rather independent of modification time. In turn, the elastic recovery decreased slightly with the increasing modification time.

To observe the effect of the rubber on the properties of bitumen, 8 h modification without the addition of rubber was also carried out. The basic properties of bitumen modified in this way are presented in Table 3. Though the lack of rubber does not affect the penetration value, it is significant for the Fraass break point and elastic recovery. The sample without rubber had at least a 15% lower elastic recovery and was less resistant to low temperatures by 2–4 °C. Moreover, the softening point of a sample without rubber indicates that it becomes softer and less viscous much faster compared to asphalt containing rubber.

**Figure 3:**
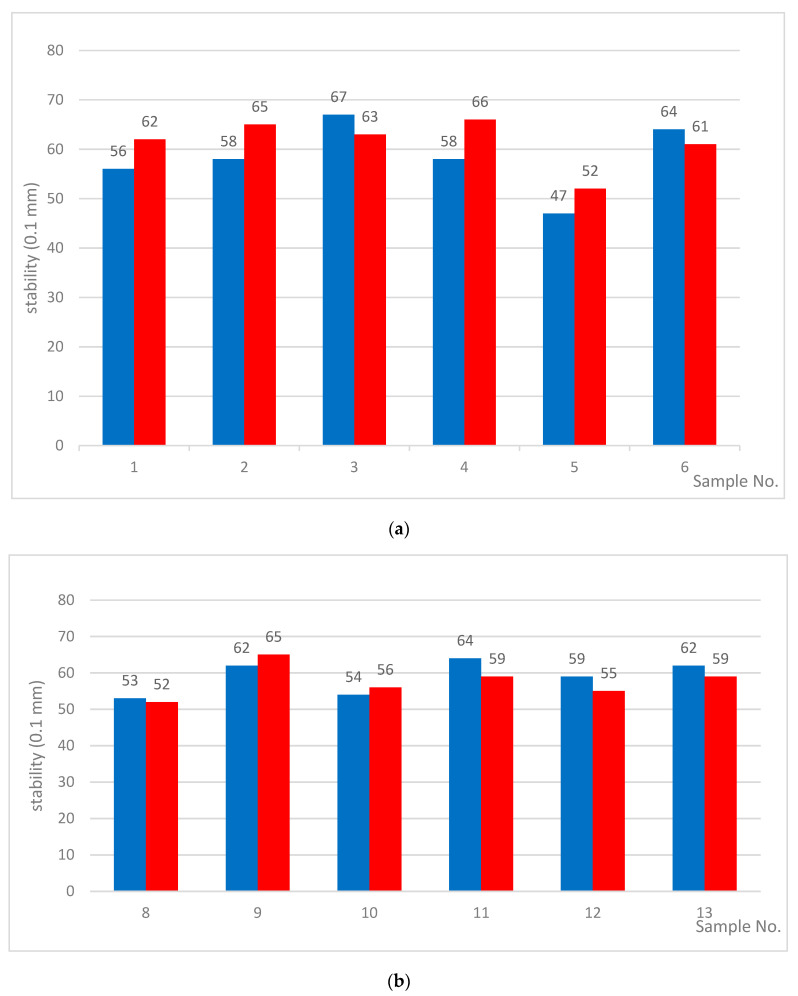
Relations between the penetration top/penetration bottom for the bitumens modified at: (**a**) 190 °C and (**b**) 220 °C.

**Figure 4:**
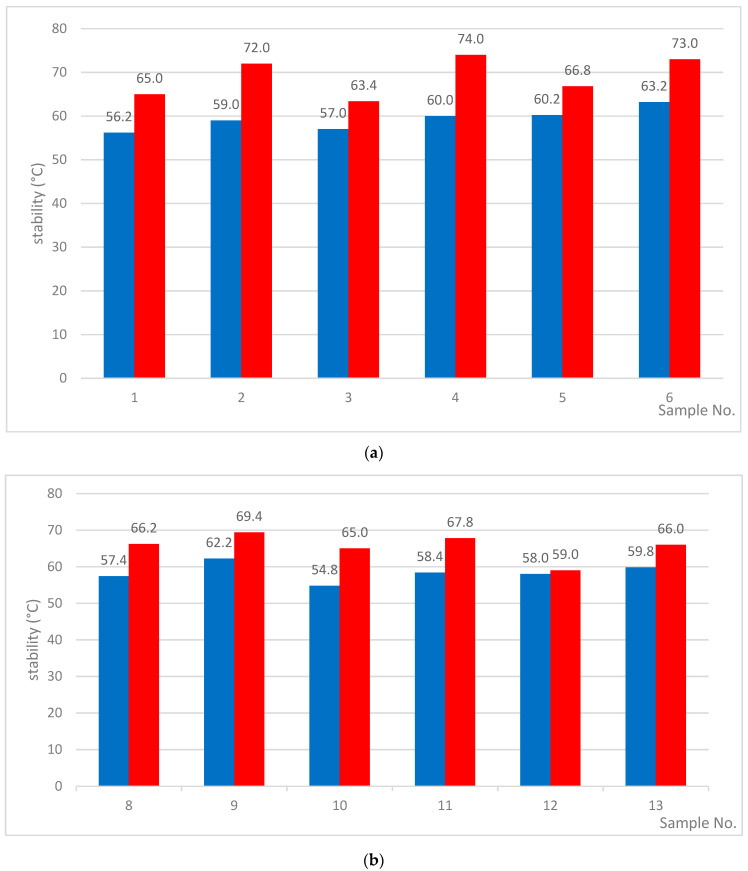
Relations between R&B top/R&B bottom for the bitumens modified at: (**a**) 190 °C and (**b**) 220 °C.

For the samples modified at 220 °C (Table 4), penetration had a more regular tendency to decrease. The softening points also became lower as the modification time increased. It is worth noting that the Fraass breaking points were even slightly lower. Regardless of the modification time, the breaking points for all tested samples were in the range of −18 °C to −20 °C. This indicates that the modification at 220 °C promotes an increase of resistance to low temperatures. At this temperature, the degree of rubber degradation was greater than at 190 °C. As a consequence, a more homogeneous structure is created. Additionally, delta penetration and retained penetration values after RTFOT for asphalt modified at 220 °C confirmed the appearance of a homogeneous phase. Due to the homogeneous structure, such bitumens retain their viscoelastic properties at low temperatures and do not flow at higher temperatures under prolonged loading. In the presented approach, no unfavorable phenomenon of asphalt phase separation was observed [27]. Independently of modification time, the obtained asphalts exhibited resistance to aging. Such properties are particularly important from the point of view of storage and distribution [29]. The results of the studies of their storage stabilities are presented in Figure 3 and Figure 4.

The values of the penetration index (PI) are also very promising. The PI expresses a quantitative measure of bitumen response to temperature changes [30]. When the asphalt penetration index is known, the behavior of such asphalt in a specific application can be predicted. The PI value should be from about −3 for high temperature bitumen to about +7 for high temperature sensitive (high PI) bitumen. For all samples modified at 220 °C, their values were in the range 0.6–1.7 and insignificantly decreased with the increasing modification time. It is important that the mass change after RTFOT for this series of samples was very small. The loss of mass, and more precisely the change of bitumen mass, is determined to check whether its density does not change as a result of oxidation or evaporation of volatile solvents [31]. For the bitumens modified at 190 °C, they change from 0.10 to 0.04%, whereas for those modified at 220 °C, they dropped from 0.15 to 0.02%.

The additional information on the effectiveness of bitumen modification with rubber is provided by the determined values of cohesion energy and maximum tensile force. The cohesive strength of asphalt represents the deformation resistance capability of the asphalt’s molecular structure. According to Tan and Guo [32], the tensile strength of the asphalt mixture is related to cohesion. The cohesion energies at 5 °C and 10 °C determined for the samples containing 10% of rubber and modified at 190 °C had constant values. The values 4.8 J/cm^2^ at 5 °C and 2.6 J/cm^2^ at 10 °C were independent of modification time. For the samples containing 15% rubber at 5 °C, breaking was observed. Cohesion energy determination was possible only for the longest-modified sample. For a larger amount of rubber, a longer time is probably needed to form a more homogeneous mixture. Such behavior confirms the results of the maximal tensile force determinations. For the samples modified at 190 °C, large fluctuations of tensile force values could be found. For the samples modified at 220 °C, such a phenomenon was not observed. In this case, changes in the maximal tensile forces were more regular. While for the samples containing 10% rubber, the values were independent of the modification time; for the samples containing 15% rubber, they increased slightly with the increasing modification time. Additionally, the values of cohesion energies at 5 °C and 10 °C were rather constant and independent of modification time. 

These data show that the addition of rubber and modification temperature have a significant impact on the quality of bitumen. The process in which instead of using rubber as a bitumen filler, the rubber de-vulcanized and then co-vulcanized with SBS led to a homogeneous product. The most homogeneous and spatially stable structures were obtained when 10% of rubber was added at 220 °C. It seems that a modification time of 2 h is sufficient. 

It should be emphasized that these bitumens contained only 2% SBS and the rubber was responsible for their properties.

## 4. Conclusions

It is possible to replace part of the SBS with a waste product such as recycled rubber during the production of polymer modified bitumen. To achieve this, the rubber must be converted from a cross-linked polymer into a thermoplastic polymer in the process of de-vulcanization. The de-vulcanized rubber was next used to create the joint net with SBS while cross-linking. The de-vulcanization and cross-linking were carried out at different times at temperatures of 190 °C and 220 °C. The co-vulcanization process carried out at 220 °C with the 10% rubber content was very fast, whereas modification at 190 °C required a longer time. As a result, products that meet the standards (PMB 45/80-55 PG 70-22) according to AASHTO-MP1, were obtained. Almost all functional properties of bitumens modified in this way reached the values for PMB. Particularly noteworthy were their high elasticity, high softening point, and favorable low-temperature properties. This means that for road building purposes, the waste product of recycled rubber can partially replace much more expensive SBS.

## 5. Patents

Gawdzik Barbara, Matynia Tadeusz. Bitumen modifier and method of bitumen modification with its use. Polish Patent No. 226748 (2017).

## Figures and Tables

**Table 1:** Bitumen modified at 190 °C.

Sample No.	Modifiers (%)			Modification Time (h)	Residue after Devulcanization (%)
	Rubber 0.4	SBS	Donor S		
1	10	2	0.07	2	5.6
2	15	2	0.07	2	12.7
3	10	2	0.07	4	5.2
4	15	2	0.07	4	9.0
5	10	2	0.07	8	5.0
6	15	2	0.07	8	6.4
7	-	2	0.07	8	-

**Table 2:** Bitumen modified at 220 °C.

Sample No.	Modifiers (%)			Modification Time (h)	Residue after Devulcanization (%)
	Rubber 0.4	SBS	Donor S		
8	10	2	0.07	2	3.6
9	15	2	0.07	2	5.0
10	10	2	0.07	4	4.0
11	15	2	0.07	4	5.0
12	10	2	0.07	8	3.3
13	15	2	0.07	8	4.5

**Table 3:** Properties of bitumen modified at 190 °C.

Property	Sample No.						
	1	2	3	4	5	6	7
penetration at 25 °C (0.1 mm)	50.4	50.6	50.0	54.0	47.0	64.0	502
softening point (R&B) (°C)	65.9	64.4	64.0	73.8	626	70.0	57.0
penetration index	1.9	2.3	1.7	2.6	1.4	2.5	-
Fraass breaking point (°C)	−17	−18	−18	−18	−18	−16	−14
elastic recovery at 25 °C (%)	75.0	80.0	75.0	76.3	76.0	76.1	58.8
stability (values of penetration top/penetration bottom) (0.1 mm)	56/62	58/65	69/63	58/66	47/52	64/61	-
delta penetration (difference between top and bottom of the tube) (0.1 mm)	4	7	6	8	5	3	-
stability (R&B top/R&B bottom)	56.2/65.0	59.0/72.0	57.0/63.4	60.0/74.0	60.2/66.8	63.2/73.0	-
delta R&B (°C)	8.8	13.0	6.4	14.0	6.6	9.8	-
softening point after RTFOT (°C)	66.0	70.2	65.6	76.6	66.8	70.4	-
increasing of softening point R&B after RTFOT (°C)	0.1	5.8	1.6	2.8	4.2	0.4	-
mass change after RTFOT (%)	+0.02	−0.10	−0.05	−0.04	+0.04	+0.09	-
cohesion energy at 5 °C (J/cm^2^)	4.8	breaking	4.8	breaking	4.8	4.3	-
cohesion energy at 10 °C (J/cm^2^)	2.6	1.9	2.5	2.0	2.6	2.4	-
maximal tensile force at 5 °C (N)	54.3	39.5	32.0	9.6	59.0	32.4	-
maximal tensile force at 10 °C (N)	26.8	18.8	16.0	6.6	30.0	18.0	-

**Table 4:** Properties of bitumen modified at 220 °C.

Property	Sample No.					
	8	9	10	11	12	13
penetration at 25 °C (0.1 mm)	53.8	61.0	50.3	59.5	50.1	57.8
softening point (R&B) (°C)	60.8	71.0	59.4	64.0	59.0	59.2
penetration index	1.4	1.7	0.9	1.7	0.6	1.2
Fraass breaking point (°C)	−18	−20	−18	−18	−19	−18
elastic recovery at 25 °C (%)	74.8	78.9	78.2	71.3	76.6	75.0
stability (values of penetration top/penetration bottom) (0.1 mm)	53/52	6/65	54/56	64/59	59/55	62/59
delta penetration (difference between top and bottom of the tube) (0.1 mm)	1	3	2	5	4	3
stability (R&B top/R&B bottom)	57.4/66.2	62.2/69.4	54.8/65.0	58.4/67.8	58.0/59.0	59.8/66.0
delta R&B (°C)	8.8	7.2	10.2	9.4	1.0	6.2
softening point after RTFOT (°C)	64.4	73.7	66.4	70.2	58.0	66.4
increasing of softening point R&B after RTFOT (°C)	3.6	2.7	7.2	6.2	7.6	5.8
mass change after RTFOT (%)	−0.07	−0.11	−0.10	−0.15	−0.02	−0.04
cohesion energy at 5 °C (J/cm^2^)	3.5	3.0	3.7	2.5	3.6	2.7
cohesion energy at 10 °C (J/cm^2^)	2.1	1.4	1.9	1.4	1.9	1.5
maximal tensile force at 5 °C (N)	53.0	30.5	52.6	37.5	54.0	37.0
maximal tensile force at 10 °C (N)	26.0	15.9	25.0	18.0	25.0	17.2
